# Xanthomatous pleuritis in a child with recurrent pneumonia and patent ductus arteriosus: A rare pediatric case report and literature review

**DOI:** 10.1016/j.rmcr.2026.102445

**Published:** 2026-06-18

**Authors:** Seyedeh Zalfa Modarresi, Atossa Madanipour, Fariba Binesh, Somayeh Talaeepour, Mahla Towhidifar

**Affiliations:** a Children Growth Disorder Research Center, Comprehensive Research Institute for Maternal and Child Health, Shahid Sadoughi University of Medical Sciences, Yazd, Iran; b Department of Pediatrics, Shahid Sadoughi Hospital, School of Medicine, Shahid Sadoughi University of Medical Sciences, Yazd, Iran; c Student Research Committee, Alborz University of Medical Sciences, Karaj, Iran; d Department of Pathology, Shahid Sadoughi University of Medical Sciences, Yazd, Iran; e Student Research Committee, Shahid Sadoughi University of Medical Sciences, Yazd, Iran

**Keywords:** Xanthomatous pleuritis, Pulmonary xanthoma, Pediatric pleural disease, Recurrent pneumonia, Patent ductus arteriosus, Pleural lipid laden macrophages

## Abstract

**Background:**

Xanthomatous pleuritis is an exceptionally rare benign inflammatory condition characterized by aggregates of lipid-laden macrophages within the pleura. To the best of our knowledge, only three adult cases have been reported worldwide, associated with chronic pleural inflammation or infection. Pediatric pleural xanthomas are exceedingly uncommon, and xanthomatous pleuritis has not been previously reported in a child.

**Case presentation:**

We report a 3.5-year-old girl with recurrent pneumonia, chronic respiratory symptoms, and Failure to Thrive (FTT). Chest X ray (CXR) revealed cardiomegaly and subpleural nodules, followed by high resolution Computed Tomography scan (CT scan) showing patchy right-sided consolidation, mild pleural thickening, subpleural nodules and loculated effusion. Diagnostic thoracentesis was performed; however, only a minimal amount of loculated fluid was obtained.

CT-guided biopsy of a pleural-based nodule demonstrated numerous lipid-laden (foamy) macrophages with scattered multinucleated histiocytes, without granuloma or malignancy, supporting the diagnosis of xanthomatous pleuritis.

Further evaluations for tuberculosis, cystic fibrosis, and primary immunodeficiency were negative. Echocardiography identified a patent ductus arteriosus (PDA). The child improved clinically with intravenous (IV) broad-spectrum antibiotics and then after Amoxicillin-Clavulanate therapy. She later underwent successful PDA closure.

**Conclusion:**

To the best of our knowledge, this case represents the first pediatric case with xanthomatous pleuritis described in the literature and emphasizes the importance of histopathologic evaluation in children with unexplained recurrent pulmonary disease.

## 1 Introduction

Xanthomatous pleuritis is an uncommon inflammatory condition characterized by lipid-laden macrophage accumulation within the pleural tissue [1]. Although xanthomatous inflammation has been described in several organs, including the kidney, gallbladder, and female genital tract, its occurrence in the pleura is exceptionally rare [2]. The process is related to a chronic inflammatory reaction to persistent tissue injury, obstruction, or infection, ultimately leading to localized deposition of foamy histiocytes [3].

Only a very small number of xanthomatous pleuritis cases have been documented in the global literature, with approximately three reported to date, all of which occurred in adults [3]. These cases were typically associated with chronic pulmonary infections such as tuberculosis, prolonged effusions, or unresolved inflammatory processes [2]. Because of its rarity and nonspecific clinical manifestations, the diagnosis is often delayed and requires histopathological confirmation to distinguish it from granulomatous diseases, malignancy, or other chronic inflammatory disorders [1].

The occurrence of xanthomatous pleuritis in children is exceedingly unusual, and to the best of our knowledge pediatric cases have not been reported yet. [[1], [2], [3], [4], [5]]. Here, we present a 3.5-year-old child with xanthomatous pleuritis.

## 2 Case presentation

A 3.5-year-old girl, born to consanguineous parents, referred to Shahid Sadoughi Hospital (a tertiary pediatric center) with fever, respiratory distress and ill and toxic appearance without clinical improvement after 1-week IV antibiotics.

She had a history of recurrent pneumonia, chronic cough, and failure to thrive since infancy. She had been repeatedly treated with oral antibiotics, but symptoms persisted.

On admission to our center, she was febrile (38.5 °C), tachypneic, and had intercostal and subcostal retractions with oxygen saturation of 92% on room air. A systolic murmur was also detected. Laboratory findings showed leukocytosis, anemia, and elevated inflammatory markers (Table 1).

**Table 1:** Laboratory data during treatment.

Parameter	Unit	Initial	Last Follow-up	Reference Range
**CBC**				
**WBC**	×10^3^/μL	27.8	4.6	5 – 15
**RBC**	×10^6^/μL	3.77	4.15	3.9 – 5.3
**Hemoglobin**	g/dL	8.0	9.2	11 – 13.5
**Hematocrit**	%	28.6	31.8	34 – 40
**MCV**	fL	75.9	76.6	75 – 87
**MCH**	pg	21.2	22.2	24 – 30
**MCHC**	g/dL	28.0	28.9	32 – 36
**Platelets**	×10^3^/μL	733	219	150 – 450
**Neutrophils**	%	71.9	52.6	30 – 60
**Lymphocytes**	%	21.2	40.1	30 – 70
**RDW-CV**	%	16.5	18.5	11.5 – 14.5
**RDW-SD**	fL	47	51	35 – 46
**MPV**	fL	7.7	9.4	7.4 – 10.4
**PDW**	%	8.9	11.8	9 – 14
**Inflammatory Markers**				
**ESR**	mm/hr	129	62	<20
**CRP**		Positive (3+)	Negative	Negative
**Biochemistry**				
**Blood sugar (FBS)**	mg/dL	94	–	70 – 110
**Urea**	mg/dL	19	12	10 – 40
**Creatinine**	mg/dL	0.4	0.4	0.3 – 0.7
**Calcium**	mg/dL	9.3	9.3	8.6 – 10.2
**Magnesium**	mg/dL	2.20	2.20	1.7 – 2.3
**SGOT (AST)**	U/L	27	–	<40
**SGPT (ALT)**	U/L	35	–	<41
**Sodium**	mmol/L	129	136	135 – 145
**Potassium**	mmol/L	5.0	4.7	3.5 – 5.5
**Microbiology**				
**Throat culture**	—	No growth	–	Negative
**Urine culture**	—	No growth	–	Negative
**Blood culture**	—	No growth	–	Negative
**Pleural culture**	—	No growth	–	Negative
**VBG**				
**pH**	—	7.49	–	7.35 – 7.45
**pCO_2_**	mmHg	37.3	–	35 – 45
**HCO_3_**	mmol/L	27.8	–	22 – 26
**Base Excess**	mmol/L	+4.4	–	−2 to +2
**Immunology**				
**NBT (Nitro blue Tetrazolium Chloride) test**	%	92	–	>90

**Fig. 1:**
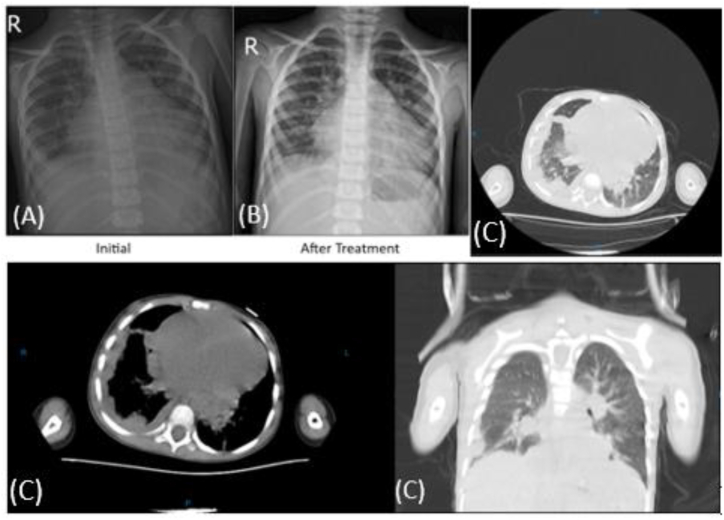
**Radiologic findings of the patient. (A)** Initial AP chest radiograph demonstrating cardiomegaly with diffuse bilateral interstitial and alveolar opacities, more prominent in the right lung. **(B)** Follow-up PA chest radiograph after 3 months showing partial resolution of pulmonary infiltrates. **(C)** Chest CT demonstrating right-sided loculated pleural effusion with pleural thickening, atelectasis, and multifocal right lung consolidation.

Chest radiograph and high-resolution CT scan demonstrated cardiomegaly, multifocal right-sided consolidations, pleural thickening, subpleural nodules and a loculated right pleural effusion (Fig. 1). Chest ultrasonography showed round lesion with internal echoes and peripheral vascularity (15 × 16 mm), minimal right pleural effusion. Diagnostic thoracentesis was performed; however, due to minimal loculated pleural fluid, only a small amount was obtained and was sent for microbiological culture.

Immunologic evaluation, sweat chloride test, and tuberculosis work-up were negative. Cultures obtained from sputum, blood, and pleural fluid showed no growth.

Echocardiography identified a small atrial septal defect (4.8 mm) and a moderate to large patent ductus arteriosus (PDA) associated with mild to moderate pulmonary hypertension.

**Fig. 2:**
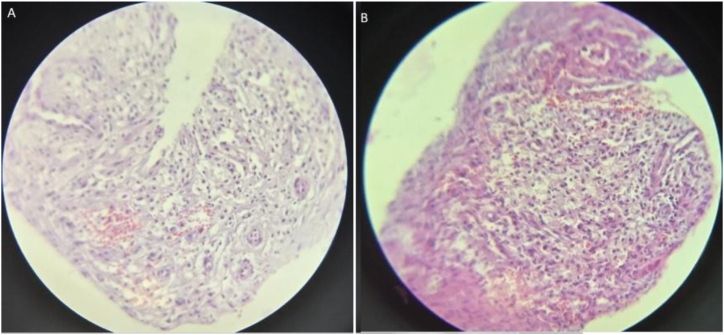
Histopathological findings of pleural biopsy. (A) Low-power view showing pleural connective tissue with diffuse chronic inflammatory infiltrate and areas of fibrosis. (B) High-power view demonstrates numerous lipid-laden (foamy) macrophages and scattered multinucleated histiocytes within the pleural stroma. No granuloma formation, necrosis, or malignant features are identified, consistent with xanthomatous pleuritis (H&E stain).

Due to persistent fever and imaging findings of a subpleural lesion, CT-guided biopsy of a right-sided pleural nodule was performed. Histopathological examination revealed pleural tissue infiltrated by numerous lipid-laden (foamy) macrophages and scattered multinucleated histiocytes within the pleural stroma consistent with xanthomatous pleuritis. No granuloma formation, necrosis, or malignant features are identified (Fig. 2).

Empiric broad-spectrum (Meropenem + Vancomycin) IV antimicrobial therapy was continued based on the overall clinical and radiologic picture suggestive of a partially treated infectious process and continued till being afebrile and inflammatory markers decreased. She was discharged with Amoxicillin Clavulanate 90 mg/kg/day and supportive medical care for pulmonary hypertension, followed by successful catheter-based PDA closure. Clinical symptoms gradually resolved, and subsequent follow-up imaging over the next year demonstrated resolution of lung lesions.

## 3 Discussion

Xanthomatous pleuritis is an exceptionally rare condition, with only three cases reported in the literature, all in adults and it is not reported in children yet [[1], [2], [3], [4], [5]]. To best of our knowledge this is the first reported case in children. Although the pathogenesis is not fully understood, most reported cases are associated with chronic pleural inflammation, recurrent infections, or mechanical pleural insults [6].

In the reported adult cases, the underlying conditions varied including chronic pleural effusion, and pancreatic pleural fistula. (Table 2). Similar to our case, Bateman et al. highlighted that chronic, unexplained pleural effusions may lead to marked pleural remodeling and prolonged inflammatory states, which create a microenvironment conducive to lipid-laden macrophage accumulation and xanthomatous transformation [4].

**Table 2:** Reported cases of xanthomatous pleuritis.

Citation (Author/Year)	Age/Sex	Clinical Context/Presentation	Predisposing Condition/Associated Finding	Treatment/Management	Outcome/Follow-up
Current case	3.5 F	Recurrent pneumonia since infancy, chronic respiratory distress, failure to thrive; subpleural nodules on chest X-ray/CT	Patent ductus arteriosus (PDA) causing recurrent pulmonary infections; mild cardiomegaly	IV Meropenem + Vancomycin + outpatient Amoxicillin-Clavulanate (90 mg/kg/day) + supportive care; catheter-based PDA closure	clinical and radiologic improvement after PDA closure
Singh & Pattari, 2018 [1]	21 M	Progressive dyspnea and dull chest pain over 3 months; left-sided pleural effusion	Chronic pleural effusion of unclear etiology; no malignancy or granuloma; exudative pleural fluid with thickened pleura	Discontinuation of prior antitubercular therapy, empirical antibiotics (Amoxicillin for 6 weeks) & conservative management	Clinical improvement, follow-up chest X-ray after 3 months showed marked improvement/reduction in effusion
Nakashima et al., 2022 [3]	62 M	Progressive exertional dyspnea; left-sided pleural effusion on chest imaging	Chronic pancreatitis with pancreatic-pleural fistula (pancreatic duct dilatation, stones, fistulous tract to pleura)	Thoracoscopic pleural biopsy & drainage, then distal pancreatectomy + chest tube drainage to treat fistula and prevent recurrence	Uneventful — no recurrence of pleural effusion over >4 years follow-up
McGuire et al., 2009 [7]	69 F	Large left-sided exudative pleural effusion; thoracoscopic pleural plaques mistaken for early mesothelioma	Non-malignant recurrent exudative effusion; chronic pleural inflammation without malignancy or granuloma	Medical thoracoscopy with pleural biopsy; systemic steroid therapy after diagnosis corrected to xanthomatous pleuritis	Rapid clinical recovery; no recurrence at 18-month follow-up

Although xanthomatous pleuritis is typically regarded as a chronic phenomenon requiring prolonged exposure to pleural irritants, it manifested in our case in a young child—a 3.5-year-old girl. Our patient, presented with recurrent pneumonia and FTT with underlying PDA, likely predisposed to repeated pulmonary infections. PDA, when left untreated, can lead to several complications including recurrent lower respiratory tract infections, pulmonary overcirculation, heart failure, growth failure, and increased susceptibility to chronic pulmonary inflammation, chronic lung disease [7,8]. These mechanisms may have contributed to persistent pleural irritation and chronic inflammation predisposed to xanthomatous change [9].

Management in this case focused on treating the underlying predisposing factor. Although there is no standardized treatment approach for xanthomatous pleuritis, previous reports have described the use of corticosteroids in select cases, and in one with Amoxicillin [1,10]. The patient received IV antibiotics and outpatient Amoxicillin-Clavulanate with supportive care, followed by catheter-based PDA closure. This approach led to clinical and radiologic resolution, emphasizing the importance of addressing predisposing conditions in pediatric patients, similar to adult cases [[1], [2], [3], [4], [5]]. Given the rarity of this condition, therapeutic decisions are generally individualized and guided by the underlying etiology rather than the xanthomatous process itself [1,4,10,11]. Notably, Bateman et al. emphasized that in patients with persistent pleural effusions of unclear etiology, early diagnostic thoracoscopy not only prevents diagnostic delay but may also avert irreversible pleural fibrosis that complicates xanthomatous presentations [4,12].

Histopathology remains the gold standard for diagnosis, particularly when infectious, immunologic, and metabolic evaluations are inconclusive [13,14], as in this case.

## 4 Conclusion

This case highlights the exceptionally rare occurrence of documented pediatric cases of xanthomatous pleural inflammation in a young child with recurrent pulmonary disease in the setting of congenital heart disease. Histopathologic examination remains essential for diagnosis, particularly after exclusion of infectious, immunologic, and metabolic causes. Management should be directed toward underlying predisposing conditions, and awareness of this rare entity may help avoid misdiagnosis and unnecessary interventions in pediatric patients with chronic pleural disease.

## Ethical considerations

Ethical approval for reporting this case was obtained from the Shahid Sadoughi University of Medical Sciences Ethics Committee (Approval Code: IR.SSU.REC.1405.046).

Written informed consent was obtained from the patient's legal guardian for publication of this case report and accompanying images.

## Authors’ contributions

Seyedeh Zalfa Modarresi^1,2^: Conceptualization, study supervision, patient evaluation, clinical data acquisition, and drafting of the initial case presentation.

Atossa Madanipour^3^: Literature search, data organization, manuscript formatting, assistance in drafting the introduction and case presentation sections, preparation of tables, and review of references and citation accuracy.

Fariba Binesh^4^: Pathological evaluation, interpretation of histopathology findings, data verification, and critical revision of the manuscript for important intellectual content.

Somayeh Talaeepour^1,2^: Literature review, preparation of the discussion section, manuscript editing, and overall coordination of manuscript development.

Mahla Towhidifar^5^: Data collection support, preparation figures, and review of references and citation accuracy.

## Funding

This research did not receive any specific grant from funding agencies in the public, commercial, or not-for-profit sectors.

## CRediT authorship contribution statement

**Seyedeh Zalfa Modarresi:** Conceptualization, Data curation, Formal analysis, Investigation, Methodology, Project administration, Resources, Supervision, Validation, Writing – review & editing. **Atossa Madanipour:** Conceptualization, Data curation, Formal analysis, Project administration, Visualization, Writing – original draft, Writing – review & editing. **Fariba Binesh:** Formal analysis, Investigation, Resources, Validation. **Somayeh Talaeepour:** Conceptualization, Investigation, Methodology, Supervision. **Mahla Towhidifar:** Investigation, Methodology.

## Declaration of competing interest

The authors declare that they have no known competing financial interests or personal relationships that could have appeared to influence the work reported in this paper.

## Abbreviations

CXR: Chest X-ray
CT: Computed Tomography
PDA: Patent Ductus Arteriosus
IV: Intravenous
FTT: Failure to Thrive
AST: Aspartate Aminotransferase (formerly SGOT)
ALT: Alanine Aminotransferase (formerly SGPT)
VBG: Venous Blood Gas
NBT: Nitro blue Tetrazolium Chloride test
CBC: Complete Blood Count
WBC: White Blood Cell
RBC: Red Blood Cell
MCV: Mean Corpuscular Volume
MCH: Mean Corpuscular Hemoglobin
MCHC: Mean Corpuscular Hemoglobin Concentration
RDW-CV: Red Cell Distribution Width-Coefficient of Variation
RDW-SD: Red Cell Distribution Width-Standard Deviation
MPV: Mean Platelet Volume
PDW: Platelet Distribution Width
ESR: Erythrocyte Sedimentation Rate
CRP: C-Reactive Protein
FBS: Fasting Blood Sugar
pH: Potential of Hydrogen (acidity/alkalinity)
pCO_2_: Partial pressure of Carbon Dioxide
HCO_3_: Bicarbonate
